# Comprehensive Analysis of Aquaporin Superfamily in Lung Adenocarcinoma

**DOI:** 10.3389/fmolb.2021.736367

**Published:** 2021-10-11

**Authors:** Guofu Lin, Luyang Chen, Lanlan Lin, Hai Lin, Zhifeng Guo, Yingxuan Xu, Chanchan Hu, Jinglan Fu, Qinhui Lin, Wenhan Chen, Yiming Zeng, Yuan Xu

**Affiliations:** ^1^ Department of Respiratory Pulmonary and Critical Care Medicine, The Second Affiliated Hospital of Fujian Medical University, Quanzhou, China; ^2^ Respiratory Medicine Center of Fujian Province, Quanzhou, China; ^3^ The Second Clinical College, Fujian Medical University, Fuzhou, China; ^4^ Department of Epidemiology and Health Statistics, Fujian Provincial Key Laboratory of Environment Factors and Cancer, School of Public Health, Fujian Medical University, Fuzhou, China

**Keywords:** lung adenocarcinoma, aquaporin family, prognosis, bioinformatics analysis, RNA sequencing

## Abstract

**Background:** Lung adenocarcinoma (LUAD) is the most predomintnt lung cancer subtype with increasing morbidity and mortality. Previous studies have shown that aquaporin (AQP) family genes were correlated with tumor progression and metastasis in several kinds of malignancies. However, their biological behaviors and prognostic values in LUAD have not been comprehensively elucidated.

**Methods:** RNA sequencing and real-time reverse transcription PCR (RT-PCR) were used to assess AQP1/3/4/5 gene expressions in LUAD patients using GEPIA and UALCAN databases. And then Kaplan–Meier analysis, cBioPortal, Metascape, GeneMANIA, TISIDB, and TIMER were utilized to determine the prognostic value, mutation frequency, and immune cell infiltration of AQP family members in LUAD.

**Results:** We found that AQP3 expression was significantly elevated and AQP1 expression was markedly reduced in LUAD patients, whereas the expression levels of AQP4 and AQP5 exhibited no significant changes. The Kaplan–Meier survival analysis indicated that the higher expressions of AQP1/4/5 were related to longer overall survival (OS). Of interest, AQP3 was significantly correlated with the clinical tumor stage and lower AQP3 expression showed favorable prognosis in stage I LUAD patients, which indicated that AQP3 may be a potential prognostic biomarker for patients. Through functional enrichment analysis, the functions of these four AQPs genes were mainly involved in the passive transport by aquaporins, water homeostasis, and protein tetramerization. Moreover, AQP1/3/4/5 expression was strongly associated with tumor-infiltrating lymphocytes (TILs) in LUAD.

**Conclusion:** AQP3 can be used as a prognosis and survival biomarker for stage I LUAD. These findings may provide novel insights into developing molecular targeted therapies in LUAD.

## Introduction

Lung cancer is known to be the primary cause of cancer deaths worldwide (Sung et al., 2021). Lung adenocarcinoma (LUAD), as one kind of non–small-cell lung cancer (NSCLC), is the most common histological type of lung cancer, with an increasing morbidity and mortality over the last few decades (Liu et al., 2014). A previous study has revealed that LUAD patients have a poor outcome in clinical practice with only 15% of 5-year survival rate (Chen et al., 2016). Therefore, to improve the prognosis of LUAD patients, it is critical to identify LUAD-associated genes and their potential mechanisms during the progression of disease.

Aquaporins (AQPs) are a family of water channels proteins which selectively mediate water transport across membranes in specific cell types in different organs and tissues (Verkman, 2012; Li and Wang, 2017). To date, 14 classes of AQPs have been identified (AQP0 to AQP12B) in mammals, and most of them have been well-characterized (Ishibashi et al., 2009; Finn et al., 2014). However, only four classes of the AQP members (AQP1, AQP3, AQP4, and AQP 5) have been identified to be expressed in lung tissues (Liu et al., 2005; Yadav et al., 2020). In previous studies, the four AQP family members were found to be widely distributed in multiple lung cell types including alveolar epithelia, which were related to microvasculature endothelia, airway epithelia, and submucosal glands (Borok and Verkman, 2002; Verkman, 2007). Moreover, these four AQP genes have been found to be expressed in NSCLC tissues and be closely related to tumor progression, invasion, and metastasis (Warth et al., 2011; Xia et al., 2014; Yadav et al., 2020). For instance, the expression of AQP3 was reported to be upregulated in NSCLC and knockdown of its expression could suppress tumor growth and prolong survival of patients (Xia et al., 2014). AQP4 was reported to be poorly expressed in LUAD, and its overexpression could suppress cell invasion and migration of LUAD (Wu et al., 2021). However, the underlying mechanism by which the four AQP family members are activated or depressed and their association with clinicopathologic features and prognosis remains unclear.

As generally known, the study of biological mechanisms based on bioinformatics analysis is one of the most important methods in cancer research. Therefore, on the basis of the analyses of thousands of gene differential expressions (DEs) or variations in copy numbers (CNVs) published online, we analyzed the expressions and mutations of AQP family members in patients with LUAD in details to determine the expression levels, underlying biological functions, and distinct prognostic values of the four AQP family members in LUAD patients.

## Materials and Methods

### Ethics Statement

This study was approved by the Academic Committee of the Second Affiliated Hospital of Fujian Medical University, and it was performed according to the principles expressed in the Declaration of Helsinki. The study received ethics approval, and all patients gave written informed consent. Additionally, the public datasets used in this study were retrieved from the published literature.

### GEPIA Data Analysis

Gene expression profiling interactive analysis (GEPIA, http://gepia.cancer-pku.cn/) is a newly developed online analytical tool, which is based on the sequencing database containing of 9,736 tumors and 8,587 normal samples from The Cancer Genome Atlas (TCGA) and the Genotype-Tissue Expression (GTEx) programs. It provides key interactive and customizable functions including tumor/normal expression profiling and differential expression analysis according to cancer types or pathological stages, patient survival analysis, similar gene detection, correlation analysis, and dimensionality reduction analysis (Tang et al., 2017).

### UALCAN Data Analysis

UALCAN (http://ualcan.path.uab.edu) is a comprehensive web portal that facilitates in‐depth analysis of TCGA gene expression data (Chandrashekar et al., 2017). In this study, we analyzed the expression of AQP genes across normal and LUAD tissues based on different tumor stages. Statistical significance was tested using Student’s t test, with the significance accepted at *p* < 0.05.

### RNA-Seq Data Analysis

Total RNA was extracted from 10 paired stage I LUAD tissues and paracancerous tissues using the RNeasy Mini Kit (Qiagen, Germany) according to the manufacturer’s protocol. Then ribosomal RNA (rRNA) was removed using the RiboZero rRNA removal kit. The rRNA-depleted RNA was fragmented and reverse-transcribed. mRNA sequencing libraries were prepared using the VAHTS total RNA-seq Library Prep kit for Illumina (Vazyme NR603, China) following manufacturer’s instructions. Differential expression analysis for mRNA was performed using DESeq2 R package (https://bioconductor.org/packages/release/bioc/html/DESeq2.html). Genes were considered differentially expressed and retained for further analysis with |Log2 (fold change)| (|log2FC|) ≥1 and statistical *p* value ≤ 0.05. The heat map was used to display the expressed pattern of AQP family members in LUAD patients.

### Real-Time Reverse Transcription PCR

The total RNA of 28 paired stage I LUAD (13 men and 15 women; mean age, 64.61 ± 8.83 years) and adjacent normal tissues was obtained using TRIzol as per the established protocol. The Takara PrimeScript™ RT reagent kit (Takara, Japan) was utilized to synthetize cDNA from 1,000 ng RNA. TB Green Mixture (Takara Bio, Japan) was used to conduct RT-PCR using QuantStudio™ 5 Real-Time PCR Systems (Applied Biosystems). Every sample was assessed in triplicate. GAPDH served as control.

The relative gene expression obtained from this normalization was evaluated using 2^−△△CT^ methods. Sequencing data were provided in Supplementary Table S1.

### Immunohistochemical Staining

IHC staining of LUAD tissues was performed in 5-μm sections. Paraffin-embedded sections were dewaxed and rehydrated in a series of alcohol to PBS. Endogenous peroxidase was then inactivated with 3% hydrogen peroxide at room temperature for 20 min. Then the slides were soaked in 0.1 mol/L citrate buffer (pH 6.0) and placed in an autoclave at 121°C for 3 min for antigen retrieval. After washing with PBS (pH 7.4) for 3 times, the sections were blocked with 1% BSA diluted in PBS at 37°C for 30 min, followed by incubation with primary antibody against AQP1 (1:100; Bioss, China) and AQP3 (1:200; Bioss, China) overnight at 4°C. Then the sections were incubated with the HRP-conjugated goat anti-mouse/rabbit antibody at room temperature for 1 h, followed by staining with the DAB until the appearance of brown color. Finally, the sections were counterstained with hematoxylin and mounted.

### Kaplan–Meier Survival Analysis

Kaplan–Meier analysis of four AQPs in LUAD tissues on overall survival (OS), first progression survival (FP), and post-progression survival (PPS) was performed using the online platform Kaplan–Meier plotter (https://kmplot.com/analysis/). Besides, we further displayed the OS among different tumor stages of LUAD patients using Kaplan–Meier curves and performed the log-rank (LR) test.

### cBioPortal Data Analysis

The cBioPortal (http://cbioportal.org) is a comprehensive web-based database that could visualize and analyze multidimensional genomic data of multiple kinds of tumors (Gao et al., 2013). Mutation frequencies, copy number variation (CNV), and the summary of the gene types in LUAD tissues were evaluated according to the online instructions of cBioPortal. The relationship between patients’ prognosis and gene mutation was analyzed using the tool of cBioPortal based on TCGA database, with a *p*-value < 0.05 regarded as significant.

### GeneMANIA Analysis

GeneMANIA (http://genemania.org/) is an online database, a predictive server for analyzing physical interactions, co-expression, and information strength of target genes. Using this database, we analyzed the correlations between AQP superfamily molecules and their interactive genes.

### Metascape Analysis

Metascape is a comprehensive tool for gene annotation and enrichment analysis. In the current study, we evaluated the functions of the four AQP and their co-expression genes. The threshold value was set as 0.01, and the enrichment factor of >1.5 and a minimum count of 3 were considered important. (Zhou et al., 2019).

### Immune Infiltration Analysis

To explore the specific associations of AQP family genes with immune cells, we utilized the TIMER database for analysis (https://cistrome.shinyapps.io/timer/), which is a database designed for analyzing immune cell infiltrates in multiple cancers. In this study, we analyzed AQP expression levels in LUAD, and their associations with tumor purity and infiltrating immune cells including CD4+T cells, CD8+T cells, B cells, neutrophils, macrophages, and dendritic cells. Furthermore, we employed the TISIDB database (http://cis.hku.hk/TISIDB/) for evaluating whether AQP genes was related to immune infiltration therapy and subtypes in LUAD patients.

## Results

### Expression Patterns of AQP Family Members in LUAD Patients

**FIGURE 1:**
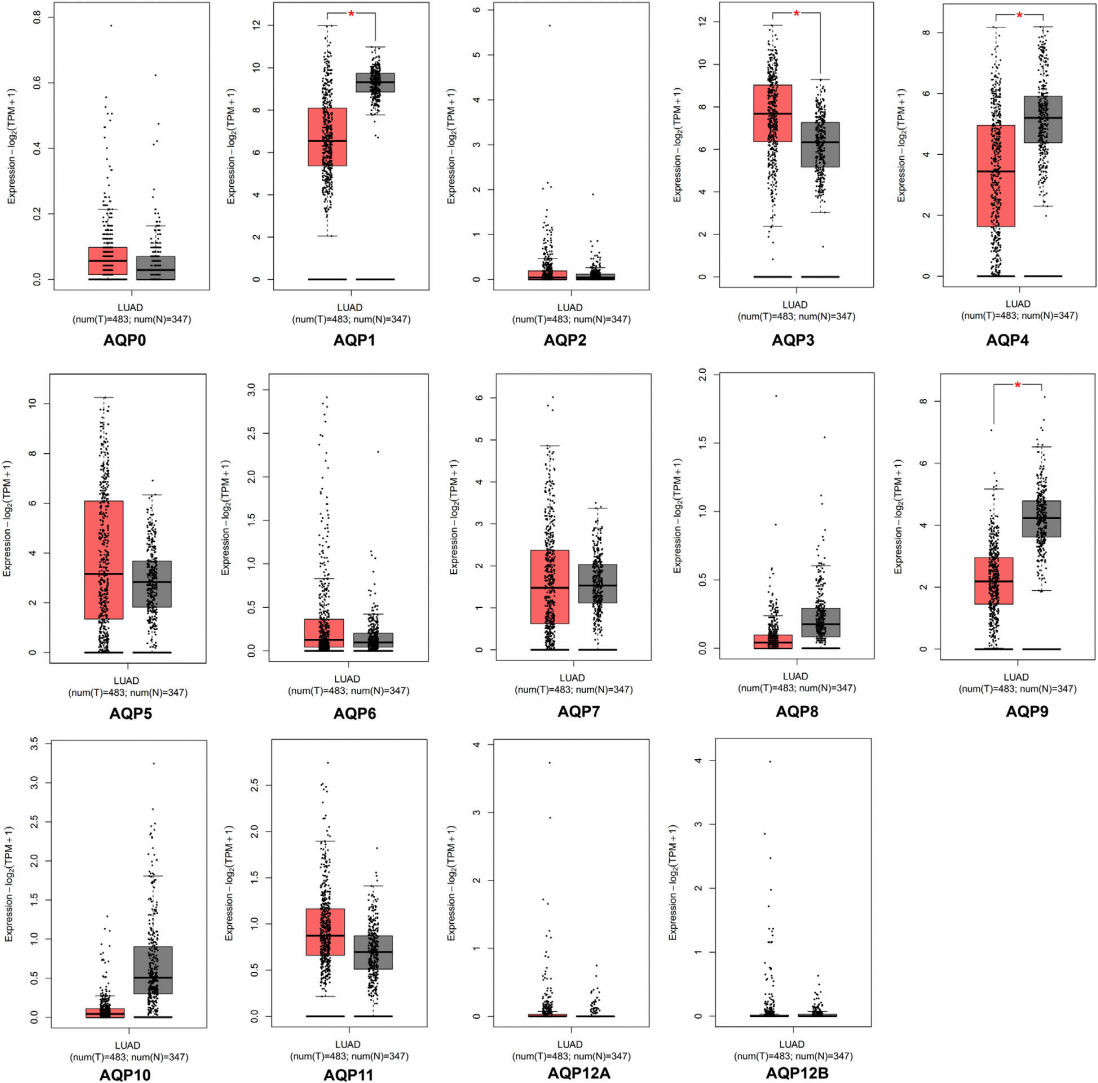
mRNA expression of AQPs family members in LUAD via GEPIA database. Red box, tumor samples; gray box, normal samples; T, tumor; N, normal (*p < 0.05).

The GEPIA database was applied to investigate the expression differences of AQP genes between tumor and normal tissues in LUAD patients at the mRNA level (tumor cases = 483, normal cases = 347). According to the GEPIA database, AQP1, AQP3, AQP4, and AQP9 were significantly expressed in LUAD tissues compared to normal lung tissues (*p* < 0.05), but some genes including AQP0, AQP5, AQP6, AQP7, AQP8, AQP10, and AQP11 were not differentially expressed between tumor and normal tissues. In addition, we found that AQP2, AQP12A, and AQP12B were not expressed either in cancerous or in normal lung tissues (Figure 1).

**FIGURE 2:**
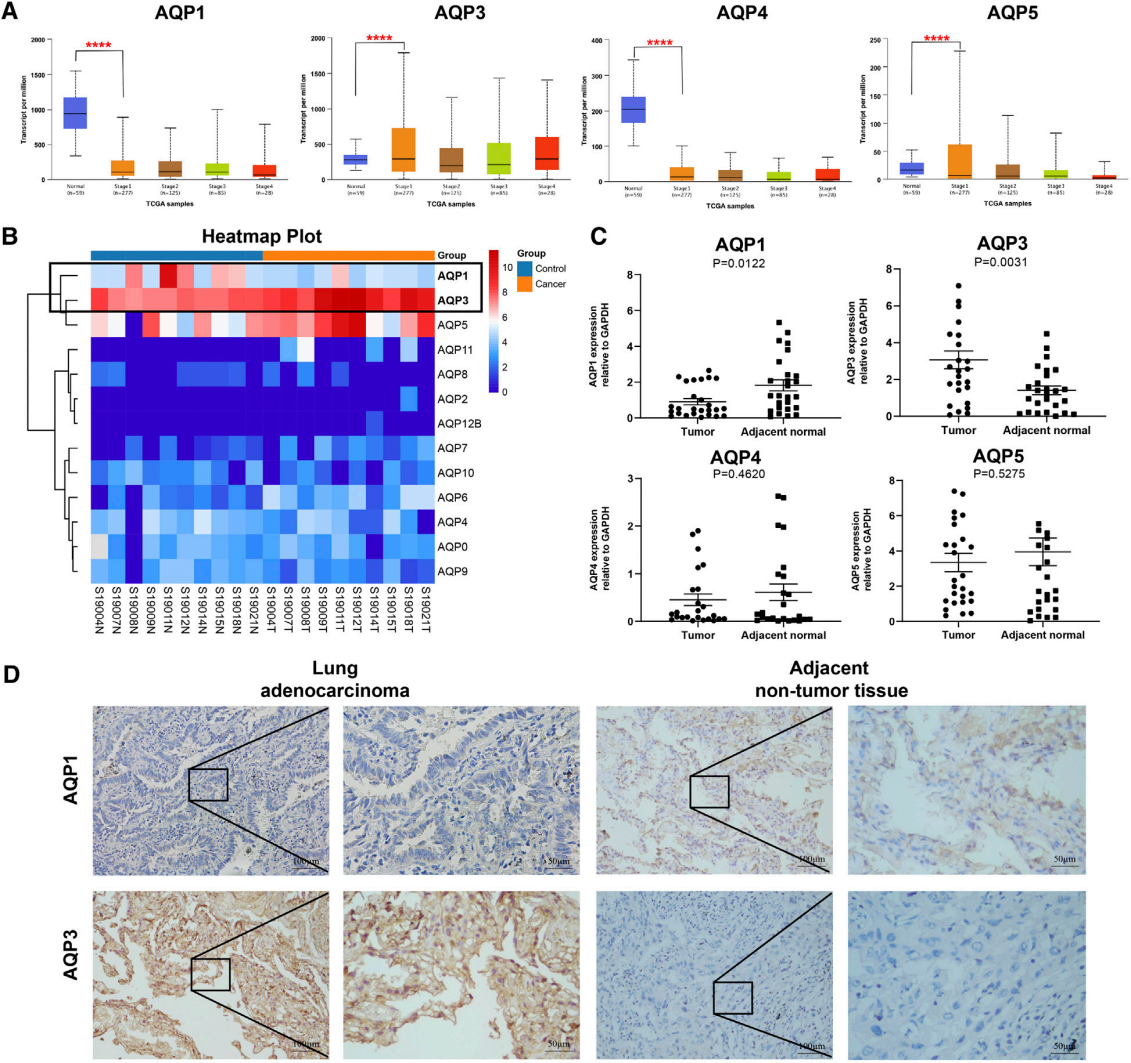
Gene expression of AQPs family members in LUAD patients. **(A)** AQP expression in LUAD patients at different clinical stages according to UALCAN. **(B)** Heat maps of AQP expression profiles identified in pulmonary normal and cancer tissues. **(C)** The mRNA expression of AQPs in LUAD tissues compared with adjacent normal tissues by RT-PCR (*n* = 28). **(D)** The protein expressions of AQP1 and AQP3 in stage I LUAD tissues compared with adjacent normal tissues via IHC (*n* = 9). (****p < 0.0001).

Previous studies have reported that four AQP family members (AQP1, AQP 3, AQP 4, and AQP 5) were expressed in lung tissues (Warth et al., 2011; Verkman, 2012; Wittekindt and Dietl, 2019); therefore, we further explored the expression levels of these genes with different tumor stages for LUAD patients using UALCAN databases. Interestingly, all these four genes were found to be aberrantly expressed in stage I LUAD patients when compared with normal tissues. As shown in Figure 2A, AQP3 was significantly increased (*p* < 0.001), while the expressions of AQP1, AQP4, and AQP5 were markedly decreased in patients with stage I LUAD (*p* < 0.001).

To further validate the expression of these four AQP family molecules in stage I LUAD, we conducted RNA sequencing analysis in 10 pairs of stage I LUAD and paracancer tissues. The heat map clearly showed an AQP-related cluster (Figure 2B), which exhibited a higher AQP3 expression and lower AQP1 expression than matched normal tissues (*p* < 0.001). Furthermore, total RNA was extracted from 28 paired stage I LUAD tissues to further validate the results of RNA sequencing and UALCAN databases by RT-PCR. The results indicated that AQP3 expression was significantly increased and the expression of AQP1 was markedly decreased in LUAD tissues compared with corresponding paracancerous tissues (*p* < 0.05) (Figure 2C). However, the expressions of AQP4 and AQP5 were not significantly different between paired tissues. Additionally, we further performed IHC staining to determine protein expression of differential AQP1 and AQP3. The results also suggested that AQP3 was highly expressed and AQP1 was poorly expressed in stage I LUAD tissues compared with paracancerous tissues (Figure 2D).

### The Prognostic Values of Four AQP Family Members in LUAD Patients

**FIGURE 3:**
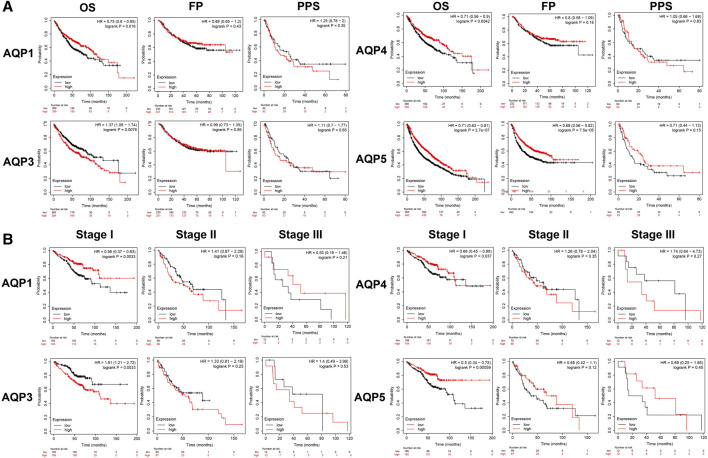
Prognostic analysis of AQP family members in LUAD patients. **(A)** Kaplan–Meier analysis of prognostic effect of AQPs with OS, FP, and PPS values in LUAD patients. **(B)** Prognostic analysis of clinicopathologic factors for OS in AQPs. OS, overall survival; FP, first progression survival; PPS, post-progression survival.

To identify the effects of AQP genes on the progression and prognosis of LUAD, the differential expression levels of these four AQP genes were associated with LUAD patients using Kaplan–Meier plotter. The Kaplan–Meier survival curve analysis revealed that higher expression levels of AQP1/4/5 were related to longer OS. Conversely, the overexpression level of AQP3 indicated a poorer OS in all LUAD patients. Beyond that, the high level of AQP5 showed a positive effect on FP. And we found that AQP1/3/4/5 expression levels had no significant correlation with PPS (Figure 3A). Next, we further analyzed the association of the expression of the four AQPs with prognosis in different tumor stages. We found that AQP1/3/4/5 expression levels showed significant statistical differences in OS among all patients with stage I LUAD. By contrast, there was no statistical significance of AQP1/3/4/5 expression linked with OS among patients with stage II and stage III LUAD (Figure 3B).

### The Alteration Frequency of Four AQP Family Genes in LUAD Patients

**FIGURE 4:**
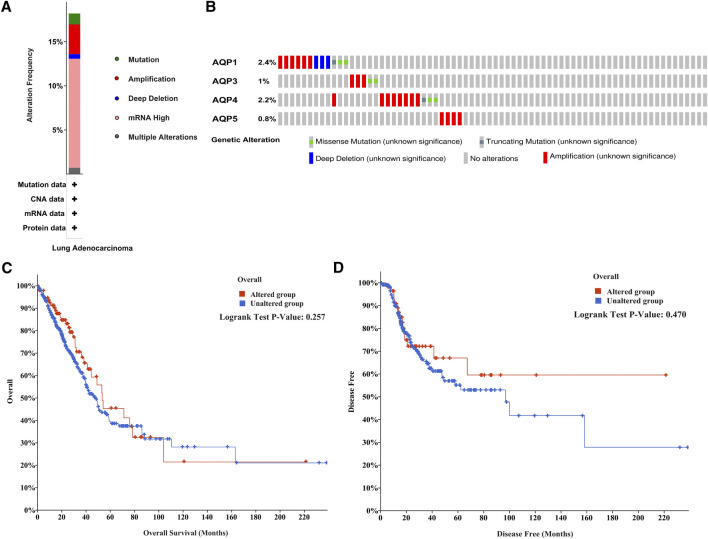
Mutation frequency of AQP family members in LUAD patients with cBioPortal. **(A)** Mutation frequency in AQP family members. **(B)** Mutation frequency in the AQP1, AQP3, AQP4, and AQP5 genes. **(C,D)** Survival analysis associated with AQP gene alterations was performed with the Kaplan–Meier plot.

And then we evaluated the frequency of genetic alterations of four AQP genes in LUAD patients using the cBioPortal database. As shown in Figure 4A, more than 15% of LUAD patients exhibited obvious alterations in the four AQP genes, including mutation, amplification, deep deletion, mRNA high expression, and multiple alterations. Besides, the genetic alterations of AQP1, AQP3, AQP4, and AQP5 among LUAD patients account for 2.4, 1, 2.2, and 0.8%, respectively (Figure 4B). Furthermore, the association between the alterations of AQP gene expression and LUAD prognosis was performed using the cBioPortal database. The results indicated that alterations of four AQP genes in LUAD patients were not significantly associated with OS (Figure 4C, *p* = 0.257) or DFS (Figure 4D, *p* = 0.470).

### Co-Expression Network and Functional Enrichment Analysis of Four AQP Family Genes in LUAD Patients

**FIGURE 5:**
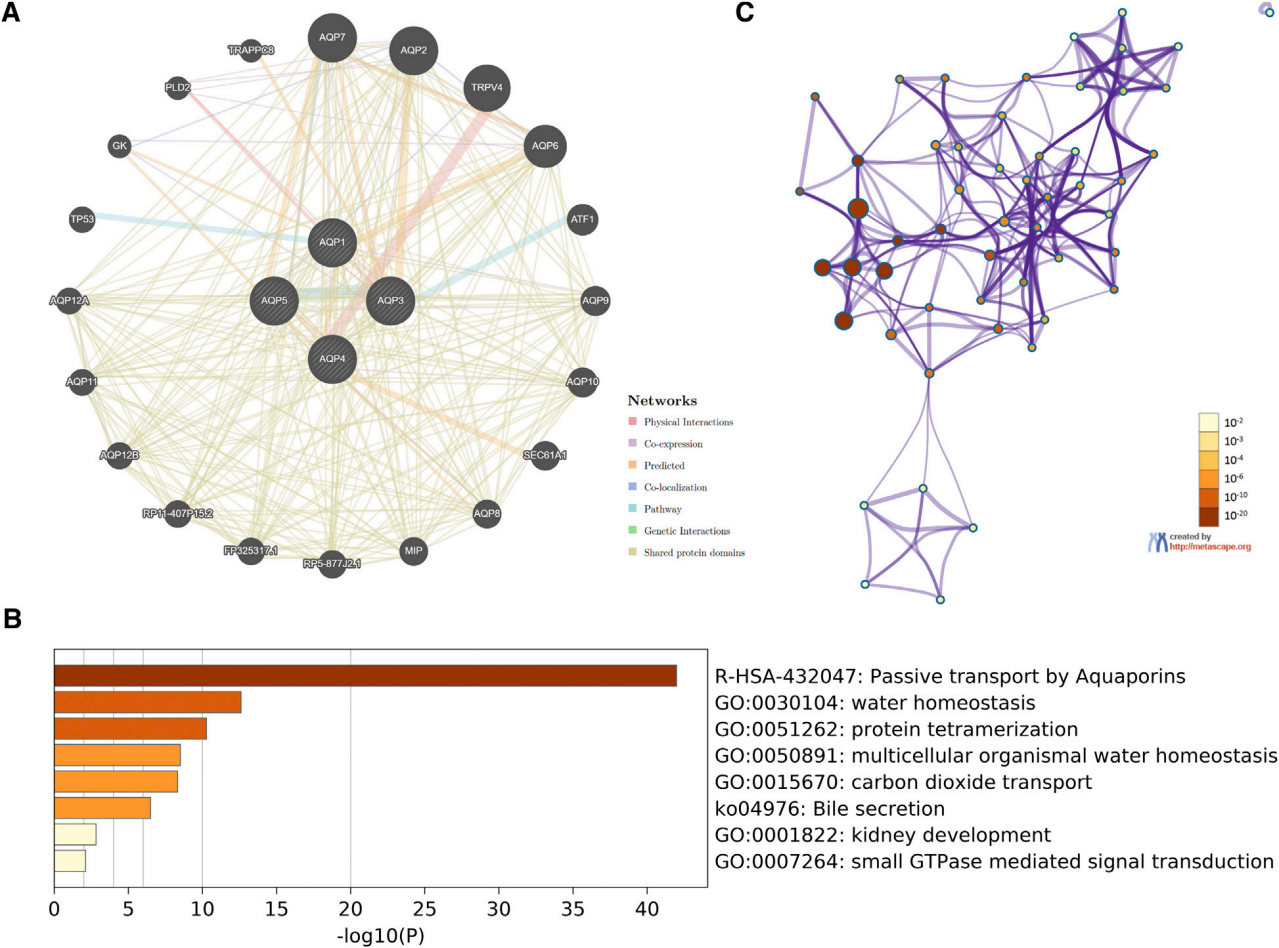
Interaction network and functional enrichment analysis of four AQP family genes in LUAD patients. **(A)** Co-expression network of AQP genes based on GeneMANIA. **(B)** Metascape analysis of top eight functional categories enriched in the AQPs. **(C)** Associations between these top eight clusters enrichment terms displayed as a network analyzed by Metascape.

In order to investigate potential mechanisms of the four AQP family genes in LUAD, the gene–gene interaction network between AQP genes and their functionally related genes was established using GeneMANIA. The results demonstrated that 20 genes, including TRAPPC8, PLD2, GK, TP53, AQP12A, AQP11, AQP12B, RP11-407P15.2, FP325317.1, RP5-877J2.1, MIP, AQP8, SEC61A1, AQP10, AQP9, ATF1, AQP6, TRPV4, AQP2, and AQP7, were mainly associated with regulatory functions of aberrantly expressed four AQP family members in LUAD patients (Figure 5A).

Next, we used Metascape for gene ontology (GO), Kyoto Encyclopedia of Genes and Genome (KEGG), and protein–protein interaction (PPI) enrichment analyses. The data revealed the eight most enriched terms, including passive transport by aquaporins, water homeostasis, protein tetramerization, multicellular organismal water homeostasis, carbon dioxide transport, bile secretion, kidney development, and small GTPase-mediated signal transduction (Figure 5B). We also constructed a network of enriched terms colored by *p*-values (Figure 5C).

### Relationship Between Four AQP Genes and Immune Cell Infiltration in LUAD

**FIGURE 6:**
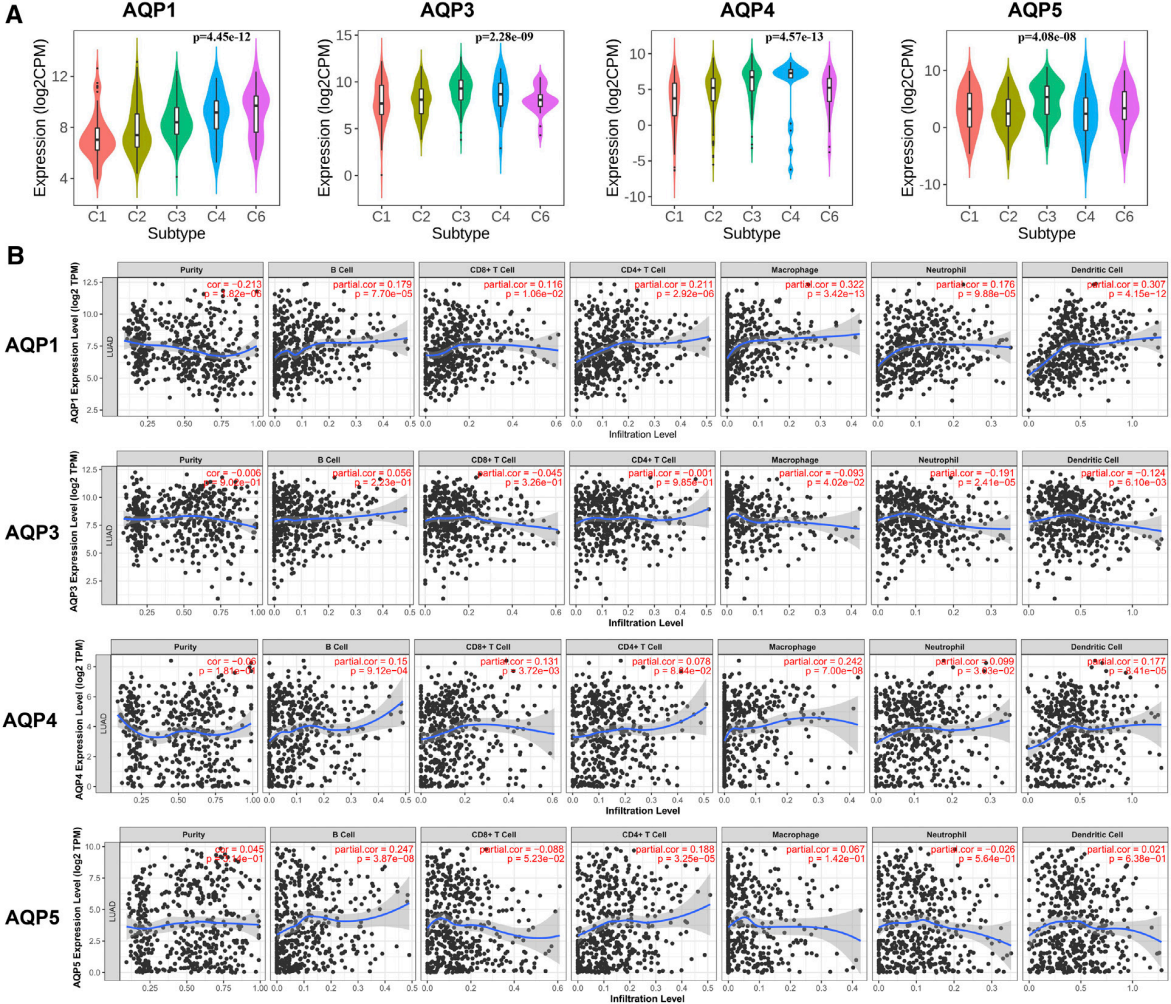
Correlations between immune infiltration and AQPs in LUAD. **(A)** TISIDB was conducted to assess the relationship between expression of four AQPs and tumor-infiltrating lymphocytes. **(B)** Relationship between AQP expression and immune infiltration level generated from TIMER.

We first analyzed the correlation between abundance of tumor-infiltrating lymphocytes (TILs) and expression of four AQP molecules using TISIDB database. Interestingly, we found that the expression levels of AQP3/4/5 were obviously increased inflammation of the LUAD immune subtype (Figure 6A, *p* < 0.001).

Next, we further evaluated the association between AQP gene expression and LUAD molecular subtypes using TIMER. Our results showed that high AQP1/4 expression and low AQP3 expression were strongly related to high infiltrating abundances of macrophages, neutrophils, and dendritic cells in LUAD, while AQP5 expression was not significantly correlated with infiltration abundances of these lymphocytes. Additionally, we found that high AQP1/5 expression was notably associated with high infiltrating abundances of B cells and CD4+ cells (Figure 6B **)**.

## Discussion

AQP family members are primarily involved in transepithelial and transcellular water flow, transport of fluid, and cell migration (Ribatti et al., 2014; Li and Wang, 2017). To date, fourteen AQP members have been identified in specific cell types in various organs and tissues; however, only AQP1/3/4/5 genes are reported to be expressed in lung tissues (Folkesson et al., 1994; Yadav et al., 2020). Additionally, several studies found that these four AQP genes were abnormally expressed in lung cancer and exerted important roles in tumor growth and cancer metastasis (Zhang et al., 2010; Warth et al., 2011), whereas more details and associations between AQPs and LUAD were not fully described. Thus, it was essential to comprehensively explore the expressions, prognostic value, and immune cell infiltration of these four AQP family genes in LUAD patients.

AQP1, which is expressed in the endothelium of the pulmonary capillary, artery, and vein (Nielsen et al., 1993), is a plasma membrane channel involved in transepithelial water transport (Verkman, 2005). Previous studies have demonstrated that AQP1 was overexpressed in lung cancer *in vitro* and *in vivo*, and upregulation of AQP1 was related to worse prognosis (Yun et al., 2016; Dajani et al., 2018; Stamboni et al., 2020). However, our study concluded contrary results. According to the GEPIA and UALCAN databases, the AQP1 expression level was significantly decreased in all LUAD patients, including stage I LUAD patients. Furthermore, the results of RNA sequencing and RT-PCR also showed a downregulated AQP1 expression in stage I LUAD. Survival curve analysis indicated that the overexpression of AQP1 was related to a better prognosis. These discrepant results may arise for several reasons, including differences in sample size, statistical methodologies, and database used. Interestingly, we found that high AQP1 expression was strongly associated with high infiltrating abundances of macrophages in LUAD. A previous study showed that functional AQP1 could exert its action in supporting M1 macrophage movement to infected regions, while repressing orientation toward the M2 phenotype (Tyteca et al., 2015). Similarly, another study supported that AQP1 suppressed M2 polarization under normal conditions, promoted M2 polarization after stimulation of LPS, and alleviated acute kidney injury by PI3K-induced macrophage M2 polarization (Liu et al., 2020a). We therefore speculated that AQP1 may also have a potentially important role for macrophages in LUAD. This speculation however requires further validation.

Unlike AQP1, AQP3 mainly expressed in basal epithelial cells, including large airway and the nasopharynx (Yadav et al., 2020). AQP3 was found to be overexpressed in lung cancer and played a critical role in tumor angiogenesis, progression, and metastasis (Xia et al., 2014; Hou et al., 2016). Wang et al. reported that AQP3 was elevated in NSCLC tissues and cells compared with the control group and partially reversed the inhibitory effects of miR‐874 on cell growth and mobility in A549 cells (Wang et al., 2020). Similarly, Liu et al. also found that AQP3 can inhibit the differentiation and apoptosis and further affect tumor progression of lung cancer stem cells by regulating the Wnt/GSK-3β/β-catenin pathway (Liu et al., 2020b). However, the associations between AQP3 expression and characteristics, prognosis, and immune-infiltrating level of LUAD patients have not been fully elucidated. In our study, we found that AQP3 was obviously upregulated in LUAD patients compared to the normal tissues in GEPIA and UALCAN databases. On further analysis of stage I LUAD patients by RNA sequencing and RT-PCR, we observed similar results. Moreover, high expression of AQP3 was significantly related to disease progression and poor prognosis in patients with LUAD. In the subgroup analysis of different tumor stages, we found that only stage I LUAD patients showed a shorter OS in the high–AQP3 expression group, which suggested that AQP3 may exert a significant role in an early stage of LUAD. Consistently, some clinical studies also suggested that the aberrant AQP3 expression may be strongly associated with tumor progression and prognosis in several malignant cancers, including hepatocellular carcinoma (Guo et al., 2013), colorectal carcinoma (Li et al., 2013), and gastric cancer (Chen et al., 2014). Therefore, AQP3 may be considered as a potential prognostic marker in patients with stage I LUAD.

AQP4 is expressed in surface columnar cells in the upper airways (Song et al., 2017), which mainly acts as the effect on facilitating fluid transport through the small airway epithelium (Zhu et al., 2016). At present, the potential functions and mechanisms of AQP4 in LUAD patients are not yet evident. Some studies showed that AQP4 was poorly expressed in LUAD tissues and overexpressing AQP4 could inhibit the promotive role of miR-196b on cancer cell migration and invasion (Wu et al., 2021), while other studies found that AQP4 was upregulated in well-differentiated LUAD and higher mRNA and protein levels of AQP4 were associated with a better prognosis (Warth et al., 2011). To validate these contradictory results, we performed bioinformatics analysis and experiments. The results showed a lower AQP4 expression in stage I LUAD patients by UALCAN database analysis, while no significant expression difference by transcriptome sequencing and RT-PCR. Furthermore, the Kaplan–Meier survival analysis revealed that higher AQP4 expression was related to a more favorable prognosis in stage I LUAD patients, which was consistent with the results quoted earlier.

Likewise, most of the studies demonstrated that AQP5 was significantly increased and played prominent roles in proliferation, migration, and angiogenesis in NSCLC (Kumari et al., 2018; Zhang et al., 2018; Elkhider et al., 2020). However, our analysis indicated that there was no differential expression of AQP5 mRNA levels in LUAD patients by bioinformatics analysis and RT-PCR, which was inconsistent with the results of previous studies (Zhang et al., 2018; Elkhider et al., 2020). In addition, we found that a high level of AQP5 showed a positive effect on OS and FP in all LUAD patients, while some researchers reported that AQP5 expression in lung cancer tissues was related to a poor prognosis (Song et al., 2015). Thus, as for these contradictory findings, further large sample size, multicentric studies and comprehensive statistical evaluation are needed to confirm.

The present study revealed the expression patterns, distinct prognostic values, and immune cell infiltration of these four AQP family genes in LUAD patients. Since our research relied on public databases and was based on bioinformatics analyses, there are some limitations. First, some results and conclusions lack experimental validation and prospectively clinical cohort validation. Second, the differences of statistical methodologies, database used, and potential sample heterogeneity may bias the outcomes. Further validation based on a larger sample size and comprehensive data analysis are required.

## Conclusion

In summary, we systematically analyzed gene expression, disease prognosis, and immune microenvironment of four AQP family members in LUAD patients. Our analysis showed that the expression level of AQP3 was significantly increased and the expression of AQP1 was markedly decreased, while the expression levels of AQP4 and AQP5 were not obviously expressed in LUAD tissues. AQP3 was found to be significantly related to the clinical tumor stage, and lower AQP3 expression indicated a better prognosis in stage I LUAD patients. These findings suggested that AQP3 could be an underlying prognosis predictor for the survival of stage I LUAD patients. Moreover, functional enrichment analysis indicated that differentially expressed AQPs is mainly involved in the passive transport, water homeostasis, and the infiltration of diverse immune cells. Thus, these findings could be a promising start for the discovery of novel potential prognosis predictors and the development of a novel target for the treatment of LUAD.

## Data Availability

The datasets presented in this study can be found in online repositories. The names of the repository/repositories and accession number(s) can be found in the article/Supplementary Material.
