# Supplementary material for: Comprehensive Analysis of Aquaporin Superfamily in Lung Adenocarcinoma
Source: Front Mol Biosci. 2021 Oct 11;8:736367. doi: 10.3389/fmolb.2021.736367 (PMC8542973; doi:10.3389/fmolb.2021.736367)
Supplement: Supplementary file 1 [file Table1.DOCX]

Supplementary Material

# Table S1. Sequence information

|  | Sequence (5’-3’) |
| --- | --- |
| AQP1 | Forward: GACCCGCTCGGACTTACT  Reverse: CTTCTGGACCCATGCTGT |
| AQP3 | Forward: CTACCTACCCCTCTGGACACTTGG |
|  | Reverse: CACGAAGACACCCGCAATGGAG |
| AQP4 | Forward: CCTCGCTGGTGGCCTTTATGAGTA |
|  | Reverse: GTCTTTCCCCTTCTTCTCCTCTCC |
| AQP5 | Forward: CGGTTCAGCCCCGCTCACTG |
|  | Reverse: CCGCTGCTCCTCCCAGTCCT |
| GAPDH | Forward: TCTACATGTTCCAGTATGACTC |
|  | Reverse: ACTCCACGACATACTCAGCACC |
